# Can Immune Response Mechanisms Explain the Fecal Shedding Patterns of Cattle Infected with *Mycobacterium avium* Subspecies *paratuberculosis*?

**DOI:** 10.1371/journal.pone.0146844

**Published:** 2016-01-25

**Authors:** Gesham Magombedze, Shigetoshi Eda, Ad Koets

**Affiliations:** 1 National Institute for Mathematical and Biological Synthesis (NIMBioS), University of Tennessee, Knoxville, TN, United States of America; 2 MRC Centre for Outbreak Analysis & Modelling, Department of Infectious Disease Epidemiology, Imperial College London, London, United Kingdom; 3 Center for Wildlife Health, Department of Forestry, Wildlife and Fisheries, University of Tennessee, Knoxville, TN, United States of America; 4 Department of Bacteriology and Epidemiology, Central Veterinary Institute, Wageningen University and Research Centre, Lelystad, The Netherlands; 5 Department of Farm Animal Health, Faculty of Veterinary Medicine, Utrecht University, Utrecht, The Netherlands; University of Illinois at Urbana-Champaign, UNITED STATES

## Abstract

Johne’s disease (JD) is a chronic disease in ruminants and is caused by infection with *Mycobacterium avium* subspecies *paratuberculosis* (MAP). At late stages of the disease, MAP bacilli are shed via feces excretion and in turn create the potential for oral-fecal transmission. The role of the host immune response in MAP bacteria shedding patterns at different stages of JD is still unclear. We employed mathematical modeling to predict if the variation in MAP shedding could be correlated to the immune response in infected animals. We used a novel inverse modeling approach that assumed biological interactions among the antigen-specific lymphocyte proliferation response (cell-mediated response), antibody/humoral immune responses, and MAP bacteria. The modeling framework was used to predict and test possible biological interactions between the measured variables and returns only the essential interactions that are relevant in explaining the observed cattle MAP experimental infection data. Through confronting the models with data, we predicted observed effects (enhancement or suppression) and extents of interactions among the three variables. This analysis enabled classification of the infected cattle into three different groups that correspond to the unique predicted immune responses that are essential to explain the data from cattle within these groups. Our analysis highlights the strong and weak points of the modeling approach, as well as the key immune mechanisms predicted to be expressed in all animals and those that were different between animals, hence giving insight into how animals exhibit different disease dynamics and bacteria shedding patterns.

## Introduction

Johne’s disease (JD) is a chronic, enteric disease in ruminants and is caused by *Mycobacterium avium* subsp. *paratuberculosis* (MAP). After infection, disease progression follows four distinct stages: (i) silent, (ii) subclinical, (iii) clinical, and (iv) advanced. In the silent stage, the animal is infected, but shedding of MAP in the feces cannot be detected [1, 2]. This stage is followed by an unpredictable but lengthy subclinical stage, the intermittent MAP shedding stage, which ranges from 2 to 10 years. The third stage (clinical stage) is evidenced by progressive wasting accompanied with reduced milk production, weight loss, and diarrhea that is correlated with fecal shedding of MAP [1]. This stage is followed by excessive MAP shedding and weight loss (the advanced stage), and then eventual death of the infected animal [3–5]. JD is costly in dairy farming because it causes reduced milk production and increased cattle mortality, and results in premature culling and reduced sale price for cattle from regions with high disease prevalence [3]. However, within the infected animal, the stages of JD appear to reflect an ongoing struggle of the host’s immune response with MAP. It is largely unclear whether antigenic load drives the immune response or whether the immune response determines the antigenic load; for instance, in tuberculosis there is a shift in understanding toward an opinion that interferon-γ (IFN-γ) responses reflect bacterial load rather than protection [6] and thus the possibility of antibody-mediated immunity (AMI).

The classical view of immune responses during MAP infection is a strong IFN-γ-driven CMI in the early stages and a low AMI response [7–11]. The predominant early CMI response is progressively lost with disease progression and is replaced with a predominantly non-protective AMI response with concomitant increased MAP fecal shedding that is observed at the subclinical, clinical, and advanced stages of the disease. As disease progresses, increasing numbers of intestinal lesions are observed. An early CMI response is reported to be associated with paucibacillary lesions [7, 12–14] that are composed of lymphocyte infiltrates, macrophages, and multinucleated giant cells with scarce acid-fast bacteria. Multi-bacillary lesions accompanied by few lymphocytes and multi-aggregates of macrophages containing numerous bacteria are observed with decreasing CMI responses and concomitant increased AMI responses [7, 12–16]. Mechanisms of how an early predominant CMI response is replaced with a predominant AMI response are still to be completely deciphered. Our recent mathematical modeling study [17] predicted several potential mechanisms for a shift in Th1/Th2 dominance, that is, (i) differential regulation of Th cell differentiation, (ii) bacteria accumulation, (iii) proliferation and differentiation inhibition of T cells, and (iv) Th1 immune exhaustion. Several other experimental studies [7, 12–14, 16] suggest that the Th1/Th2 switch could be a result of the immuno-suppressive effects of cytokines like interleukin 10 (IL-10) and transforming growth factor-β (TGF-β) on Th1 responses. It has been suggested in two studies [13, 18] that as disease progresses, there is increased suppression of Th1 responses, potentially orchestrated by IL-10 from regulatory T cells and macrophages, that could lead to reversed Th1/Th2 immune dominance.

Deciphering host immune responses following exposure to MAP and characterizing responses at different stages of infection remains a complex and a daunting task [16]. It is hypothesized that the variation of the time to shedding and the variable expression of CMI/AMI responses could be a result of variation in infective doses, temporal variations in pathogenetic events [19], and the complex dynamics of immune responses causing or responding to host transitions through the different stages of infection. Variations can arise from responses that are inherent to an individual host and due to differing virulence of the micro-organisms [20]. A study by Verna et al. [21] using lambs showed infection with different MAP C-strains to be associated with different immunopathologies and histopathology. Lesions formed in different groups of sheep [7, 14, 21] were shown to vary with intensity/dose and type of MAP strain used for infection. This led to differences in MAP bacterial counts cultured from tissues and feces. Similarly, there are variant disease outcomes following cattle exposure to MAP infection. Not all MAP-exposed animals get infected and of those that do, disease pathology is heterogeneous. This heterogeneous immunopathology is also reflected in the intermittent and unpredictable shedding patterns observed in individual infected animals. In the present study, we developed mathematical models to predict and explain potential relationships between antigen-specific lymphocyte proliferation and anti-MAP antibody production to explain the varied fecal shedding patterns observed in experimentally MAP-infected cattle. We hypothesized that the disparate MAP shedding patterns observed for each animal can be explained by the differentially expressed or suppressed immune responses within animals.

## Materials and Methods

### Statement of ethical approval

The animal experiments described in this study were performed in strict accordance with the provisions of the European Convention for the protection of vertebrate animals used for experimental and other scientific purposes (86/609 EG). The animal experiments were approved by the Animal Experiment Commission of the Central Veterinary Institute, Wageningen University and Research Centre, in accordance with Dutch regulations on animal experimentation under number 299-47053-07/99-01.

### Cattle experimental infection data

In this study, we used mathematical models to determine how the MAP pathogen interacts with host immune responses. We developed mathematical models and compared them to a dataset based on 20 cattle that were experimentally infected with MAP and followed over a period of 55 months [22].

### Animals

Twenty Holstein-Friesian calves were purchased at birth from different commercial farms and housed at specific pathogen-free facilities of the Central Veterinary Institute (CVI) in Lelystad, the Netherlands, throughout the experimental period. Experimental procedures were approved by the Ethical Committee of the CVI. Animals were kept on a regular feed regimen according to their age and lactation status, but never received fresh grass. Calves were followed over an experimental period of 55 months (from January 1, 1999 to August 27, 2003). During the course of the investigation period, 7/20 (35%) of the initial cattle survived to the end of the study. This experiment was designed to run, as closely as possible, with conditions common to Dutch dairy farming practice. Therefore, all animals were bred at 15 months of age in order for calving and milk production to start at about 2 years of age. A major cause for animals to be culled was infertility (n = 6). Cows that did not conceive were culled at about 2 years of age. Two animals were culled early following the first calving: one due to severe lameness and the other due to fatty liver syndrome. The remaining five animals were culled during the last 6 months of the study due to common disorders such as lameness and mastitis. None of the animals developed any signs of clinical JD (severe diarrhea, weight loss, emaciation, edema). In Dutch dairy herds, the average life span of a cow is just over 4 years, and losses during the current study did not exceed the losses typically observed on well managed commercial dairy farms.

### Experimental infection

Calves were infected with 20 g MAP-contaminated feces given orally, three times weekly for a period of 4 weeks during the first month of life. The inoculum was obtained from a cow with clinical signs of MAP infection: consistently shedding IS900 DNA-positive MAP. Time 0 in our experimental data denotes the day the first blood and fecal samples were taken from the calf, just prior to the first dose of oral MAP infection.

### Fecal shedding measurements

Rectal samples for fecal culture were taken approximately every 2 weeks. Bacteria were cultured according to a modified method of Jorgenson [23]. Growth of MAP was mycobactin dependent and checked every 4 weeks. If no growth was observed after 6 months of culture, the sample was considered negative. The presence of MAP in positive cultures was confirmed by amplification of MAP-specific IS900 DNA via polymerase chain reaction [24]. Shedding data was expressed semi-quantitatively in four categories: 0 = negative, 1 or + = 1–10 colony forming units (CFU)/slant; 2 or ++ = 11–100 CFU/slant; and 3 or +++ = > 100 CFU/slant.

### Blood sampling

Blood was collected from the jugular vein into heparinized tubes and serum tubes (BD Vacutainer, Becton, Dickinson and Co, Europe) in approximately 1-month intervals. Heparinized blood was used for the isolation of peripheral blood mononuclear cells (PBMC). Serum was stored at –20°C and processed at a later time point.

### Antigen

Purified protein derivative (PPD-P, Johnin) antigen was used in the lymphocyte proliferative (or stimulation) test (LPT) and the enzyme-linked immunosorbent assay (ELISA). The PPD-P was produced at CVI, Lelystad, as previously described, from MAP strains 3 + 5 and C [25].

### Cellular immune response measurements

The PBMCs were isolated and cultured according to the methods described in detail elsewhere [26]. The LPTs were performed according to methods described previously [26]; in short, cells were cultured in 96-well microtiter plates using 100 μl PBMC suspension and 100 μl antigen per well in triplicate. The PPD-P antigen was used in predetermined optimal concentrations of 10 μg/ml. Concanavalin A was used as a positive control (2.5 μg/ml) and medium alone as a negative control. Cells were cultured at 37°C and 5% CO_2_ in a humidified incubator for 3 days. Then 0.4 μCi (= 14.8 × 103 Bq) ^3^H (tritiated) thymidine (Amersham International) was added to each well, and cells were cultured for an additional 18 hours. Subsequently, cells were harvested onto glass fiber filters. Incorporation of ^3^H thymidine was measured by liquid scintillation counting and expressed as counts per minute, which was used as a measure of the intensity of antigen-specific T cell responses.

### Humoral immune response measurements

Antibodies (total immunoglobulin G) specific for PPD-P were detected by ELISA according to the method described earlier [27]. All sera were diluted 10 × in blocking buffer. Results were expressed as background corrected mean optical densities, measured at 405 nm wavelength, which was used as a measure of the intensity of humoral immune response and was denoted as ELISA.

All MAP shed in feces were measured and recorded for each animal. In our models, we used the LPT response as a proxy for the CMI, the ELISA result as a proxy for the AMI, and the fecal culture CFU results as a proxy of the within-host extracellular bacteria at the site of infection. All observed measurements were normalized by dividing them by their respective maximum value observed across all animals for each measured variable. Therefore, we fitted LPT data against the LPT (*L*) model-simulated value, antibody response against the AMI (*A*) model-generated value, and CFUs (*B*_*tot*_) against the bacterial burden model-simulated value. To reduce fluctuations in the observed data, the data was smoothed by taking a moving average over every interval of 100 days. Since CFU data was semi-quantitative, it was averaged and normalized to give a continuous profile that showed how shedding patterns evolved over time with 0 indicating no shedding and 1 indicating high shedding (+++ category), while the in-between spectrum, that is the normalized values 0 < CFUs < 1, typifies the range of shedding from low to high that correspond to the categories + (1–10 CFUs) and ++ (11–100 CFUs).

### Mathematical modeling

**Fig 1:**
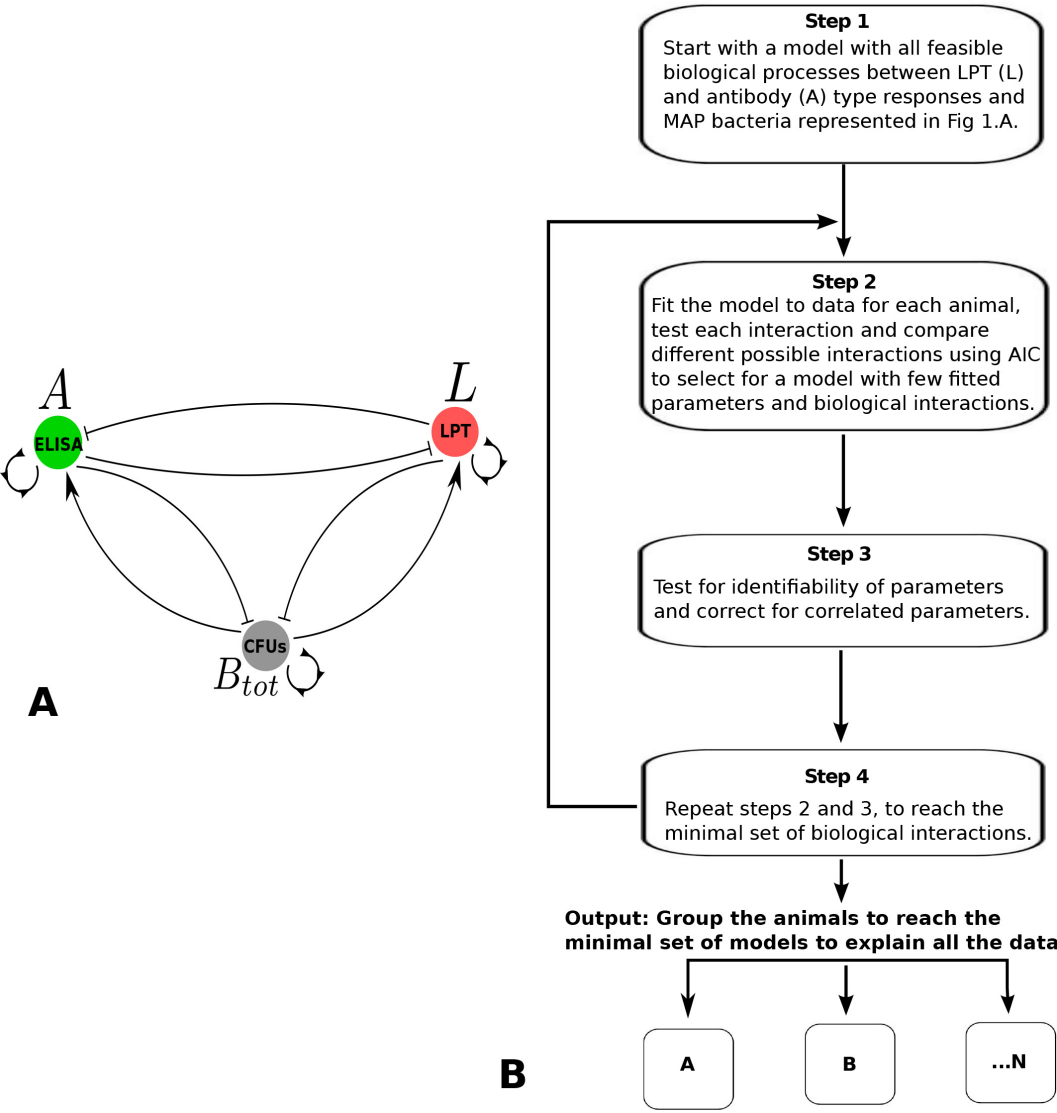
Model Cartoon and Model Selection Flowchart. A. Possible immune response interactions. Lymphocyte proliferative T (LPT) cell response data is used as a proxy for a CMI response (*L*), ELISA data is used as proxy for the antibody response (*A*), and CFU data is used as a proxy of within-host population MAP bacterial density (*B*_*tot*_) at the site of infection. Interactions represented here assume that CMI and the antibody/humoral response cross suppress and that free bacteria will drive development of both immune responses (CMI and AMI); the immune responses are assumed to suppress the MAP population. B. Illustration of the step-by-step model selection procedure used to refine the model with all the feasible biological interactions to derive the reduced minimal models with the essential biological interactions that can explain the data.

To study the fecal shedding patterns and the immune response kinetics of the infected cattle, we used a novel inverse mathematical modeling method. The approach assumed no prior knowledge of the immune pathway that is involved in controlling the way the infected animals shed MAP in feces. We then applied this approach to predict the essential immune mechanisms from a pool of several possible mechanisms by identifying and returning the minimal immune interactions that can explain the MAP shedding and immune response data of experimentally infected cattle. We started with a model that assumes all possible interactions between several immune interactions (based on CMI and AMI responses) that correlate with the observed fecal shedding patterns. We developed an iterative framework that we used to test all the potential MAP pathogen interactions with the CMI and AMI responses. We discriminated and eliminated the initially assumed/predicted immune interactions that did not help/improve the explanation of the data through fitting these interactions to data. The method seeks to predict and return only the essential minimal interactions that can reproduce the trends observed in the data. How this approach was used to select the essential biological interactions is illustrated in the top-down model selection algorithm (Fig 1) and system of Eq (1). Note that Fig 1A does not include all possible biological interactions; it is used here to demonstrate how the selection algorithm works (for a complete map of potential biological interactions, see S1 Fig).

### Top-down model selection algorithm

Given potential immune response and MAP interactions, as shown in Fig 1A, and following the steps outlined in the flowchart (Fig 1B), the top-down model selection algorithm is as follows:

1. Assume a model framework to be given by the system of Eq (1), in a way that attempts to capture all potential biological feasible interactions.
2. Fit the model interactions to data for each individual animal, testing to determine whether each interaction improves the fit using Akaike information criterion (AIC): $\text{AIC}\;=n\;\text{log}\;\left(\frac{RSS}{n}\right)+2k$, assuming identically and independently distributed errors, where *n* is the number of data points, *k* is the number of parameters fitted, and RSS is the residual sum of squares (see S1 Table for RSS and AIC values for the selected models, Table 1 for the estimated model parameters, and S2 Table for an illustration of model selection).
3. Test for identifiability of the fitted parameters for the predicted biological interactions through testing for their collinearity (multi-collinearity occurs when independent variables are so highly correlated that it becomes difficult to distinguish their individual influences on the response variable). The parameter set with the smallest collinearity index is determined by $=(1/\sqrt{min(eigenvalue({\hat{S_{ij}}}^{T}\hat{S_{ij}}))}$ where $\hat{S_{ij}}=S_{ij}/\sqrt{\sum_{j}S_{ij}^{2}}$ and *S*_*ij*_ is the normalized and dimensionless sensitivity matrix of model parameters *p*_*i*_ and model output variable *y*_*i*_. $S_{ij}=\frac{\partial y_{i}}{\partial p_{j}}\;\frac{Wp_{j}}{Wy_{i}}$ where *W*_*pj*_ and *W*_*yi*_ are the scaling constants. A parameter set with a collinearity index equal to 1 is deemed identifiable, and as the index grows, the parameter set is predicted to be linearly dependent and therefore un-identifiable (typically an index value of 20 is accepted as a cutoff of identifiability) [28].
4. Repeat steps 2 and 3 until determining the minimal biological interactions that can reproduce the kinetics of CMI (*L*), AMI (*A*), and CFUs (*B*_*tot*_) observed in the data.

**Table 1:** Estimated Parameters. A dash (-) indicates a parameter was excluded (not relevant to explain data for that animal) from the fitting procedure, and therefore the corresponding biological process was not essential in explaining the data. Parameters *β*_*1*_ and *β*_*1*_ were fixed at 0.01 during fitting using all models with data from all animals.

	Parameter Confidence Intervals							
Cattle Number by Group	*α*_1_	*α*_2_	*α*_3_	*γ*_1_	*g*_1_	*g*_2_	*h*_1_	*h*_2_
**Group A**								
01	0.0405 (0.03,0.05)	0.00021 (0.00014, 0.00025)	0.097 (0.07, 0.12)	0.3335 (0.25, 0.45)	0.4313 (0.27, 0.46)	-	-	-
05	0.0165 (0.01,0.02)	0.0102 (0.006, 0.011)	0.101 (0.07, 0.13)	0.4371 (0.29, 0.53)	0.0420 (0.03, 0.06)	-	-	-
06	0.0460 (0.03,0.06)	0.008 (0.007, 0.013)	0.1038 (0.07, 0.12)	0.1042 (0.07, 0.12)	0.060 (0.05, 0.09)	-	-	-
14	0.2097 (0.14,0.24)	0.0253 (0.015, 0.350)	0.2694 (0.20, 0.35)	0.3738 (0.29, 0.52)	0.3197 (0.22, 0.38)	-	-	-
18	0.0317 (0.02,0.05)	2.14E-10 (2.1E-10,4.7E-10)	0.2493 (0.19, 0.30)	0.7191 (0.32, 0.81)	0.6389 (0.61, 0.76)	-	-	-
19	0.1835 (0.13,0.23)	0.00071 (0.0005, 0.0009)	0.2663 (0.19, 0.34)	0.4149 (0.30, 0.52)	0.2383 (0.19, 0.32)	-	-	-
**Group B**						*g*_3_		
02	0.0166 (0.015, 0.017)	0.00075 (0.0006, 0.0008)	0.0461 (0.04, 0.05)	0.0701 (0.06, 0.08)	0.6212 (0.61, 0.68)	3.05E-6 (2.80E-6, 3.11E-6)	97.6184 (95.37, 109.47)	-
03	0.00713 (0.0070, 0.0072)	0.00312 (0.0030, 0.0032)	0.0513 (0.0511, 0.0516)	0.4993 (0.49, 0.50)	0.2760 (0.27, 0.28)	0.1548 (0.150, 0.156)	71.2519 (70.02, 72.39)	-
**04**	0.0212 (0.0204, 0.0214)	0.00624 (0.0060, 0.0065)	0.0338 (0.033, 0.035)	0.0436 (0.042, 0.045)	0.7228 (0.69, 0.75)	0.0767 (0.07, 0.08)	20.3788 (19.65, 21.23)	-
07	0.0163 (0.012, 0.021)	0.00183 (0.0013, 0.0024)	0.0957 (0.07, 0.12)	0.1269 (0.09, 0.16)	0.2363 (0.17, 0.30)	0.9999 (0.78, 1.29)	4.9160 (3.88, 6.33)	-
08	0.00375 (0.0032, 0.0040)	0.00345 (0.0027, 0.0038)	0.1019 (0.09, 0.11)	0.2557 (0.24, 0.26)	0.000103 (9.8E-5, 1.1E-4)	4.86E-10 (4.05E-10, 5.40E-10)	11.4956 (10.58, 12.42)	-
11	0.0364 (0.032, 0.039)	0.0161 (0.014, 0.019)	0.1158 (0.1121, 0.1230)	0.4376 (0.42, 0.45)	3.98E-11(2.28E-11, 6.47E-11)	0.8869 (0.79, 0.94)	8.9554 (8.87, 9.08)	-
13	0.0152 (0.014, 0.016)	0.0116 (0.011, 0.012)	0.0932 (0.091, 0.095)	0.2042 (0.20, 0.21)	7.35E-7(7.09E-7, 7.44E-7)	0.2672 (0.26, 0.27)	6.7616 (6.55, 6.81)	-
**16**	0.03745 (0.026, 0.038)	0.00968 (0.008, 0.011)	0.1216 (0.11, 0.14)	0.1588 (0.15, 0.21)	0.778 (0.53, 0.78)	0.00156 (0.0014, 0.0024)	14.4493 (12.86, 15.61)	-
**17**	0.04436 (0.044, 0.045)	0.00807 (0.0080, 0.0082)	0.03463 (0.034, 0.035)	0.02094 (0.02, 0.021)	1.0331 (1.02, 1.05)	0.03337 (0.033, 0.034)	0.03125 (0.031, 0.032)	-
**Group C**						*g*_2_		
09	0.171 (0.15, 0.18)	0.00706 (0.006, 0.008)	0.090 (0.080, 0.093)	0.1639 (0.16, 0.18)	0.4865 (0.44, 0.53)	0.3216 (0.28, 0.34)	99.9697 (82.02, 108.31)	1.47E-6 (1.24E-6, 1.61E-6)
10	0.49 (0.30, 0.62)	0.00165 (0.001, 0.022)	0.4184 (0.31, 0.58)	0.8400 (0.47, 0.95)	0.6458 (0.24, 0.72)	0.7828 (0.31, 1.00)	12.04 (9.02, 18.83)	0.4190 (0.34, 0.79)
12	0.706 (0.64, 0.72)	0.02374 (0.023, 0.024)	0.04734 (0.039, 0.048)	0.1257 (0.09, 0.13)	0.2515 (0.184, 0.253)	0.1262 (0.09, 0.13)	98.45 (72.24, 99.56)	2.18E-8(1.68E-8, 2.22E-8)
15	0.00362 (0.0021, 0.0053)	0.00562 (0.003, 0.008)	0.1082 (0.06, 0.15)	0.3851 (0.22, 0.59)	0.04899 (0.027, 0.069)	0.02348 (0.01, 0.03)	0.5694 (0.29, 0.78)	0.00143 (0.0008, 0.0020)
20	0.0266 (0.018, 0.036)	0.02144 (0.013, 0.027)	0.2602 (0.16, 0.35)	0.6317 (0.40, 0.80)	0.01425 (0.009, 0.019)	0.7251 (0.71, 0.96)	94.50 (65.60, 122.20)	0.9999 (0.62, 1.21)

In our model (equations below), the variables *L* and *A* stand for the LPT and AMI measured using the antigen-specific LPT and anti-MAP ELISA, respectively.

$$\begin{array}{c} \frac{dL}{dt}=\alpha_{1}B_{tot}^{g_{13}}L^{g_{1}}/\left(1+h_{1}A\right)-\beta_{1}L,\\ \frac{dA}{dt}=\alpha_{2}B_{tot}^{g_{23}}A^{g_{2}}/\left(1+h_{2}L\right)-\beta_{2}A,\\ \frac{dB_{tot}}{dt}=\alpha_{3}B_{tot}\left(1-B_{tot}\right),-\gamma_{1}LB_{tot}-\gamma_{2}AB_{tot} \end{array}$$ (1)

The system of Eq (1) explains the interactions represented in Fig 1A. The MAP bacteria (*B*_*tot*_) are assumed to stimulate the expression of both LPT (L) and antibody (A) responses at different rates, as represented by the parameters in the exponents *g*_*13*_ and *g*_*23*_, respectively. The *L* and *A* responses are assumed to cross suppress each other through the terms 1/(1 + *h*_1_*A*) and 1/(1 + *h*_2_*L*), respectively, where *h*_*1*_ is the suppression parameter of the *L* response on the *A* response and *h*_*2*_ represents the suppression of *A* by *L*. Production (stimulation, up-regulation, or proliferation) of *L* and *A* response-related cells is modeled by the terms $\alpha_{1}B_{tot}^{g_{13}}L^{g_{1}}/\left(1+h_{1}A\right)$ and $\alpha_{2}B_{tot}^{g_{23}}A^{g_{2}}/\left(1+h_{2}L\right)$, where *g*_*1*_ and *g*_*2*_ models the stimulation of the immune responses. A positive exponent represents stimulation while a negative represents suppression. The parameters in the exponent assume that the process of production could be non-linear, and through model fitting and mechanism selection, alternative mechanisms can be selected and tested to simplify the terms. For example, the first term on the right side of the equation for *L* can start with a general form $\alpha_{1}B_{tot}^{g_{13}}L^{g_{1}}\;A^{-h_{1}}$, and this term can be improved through testing several alternative mechanisms ($\alpha_{1}B_{tot}^{g_{13}}L^{g_{1}}/A^{h_{1}}\;or\;\alpha_{1}B_{tot}^{g_{13}}L^{g_{1}}/(1+A^{h_{1}})$ or $\alpha_{1}B_{tot}^{g_{13}}L^{g_{1}}/\left(1+h_{1}A\right)$ that can represent similar dynamics. In this case, the general term can be replaced with $\alpha_{1}B_{tot}^{g_{13}}L^{g_{1}}/\left(1+h_{1}A\right)$. This new term can be simplified further to *α*_1_*B*_*tot*_*L*, if *h*_*1*_ = 0, assuming *A* inhibition on *L* is not essential to explain the data, and with the assumption that production of *L* depends on the population of MAP bacteria (*B*_*tot*_) following the law of mass action, that is, *g*_13_ = *g*_1_ = 1. Similar simplifications can be done for the terms that govern the dynamics for the equation of *A*. This model frame work assumes that MAP bacteria replicate following a logistic growth function, *α*_3_*B*_*tot*_(1 − *B*_*tot*_), and *L* and *A* responses can eliminate the pathogen through the terms *γ*_*1*_*LB*_*tot*_ and *γ*_*2*_*AB*_*tot*_, respectively.

### Selected models, estimated parameters and their uncertainty

**Fig 2:**
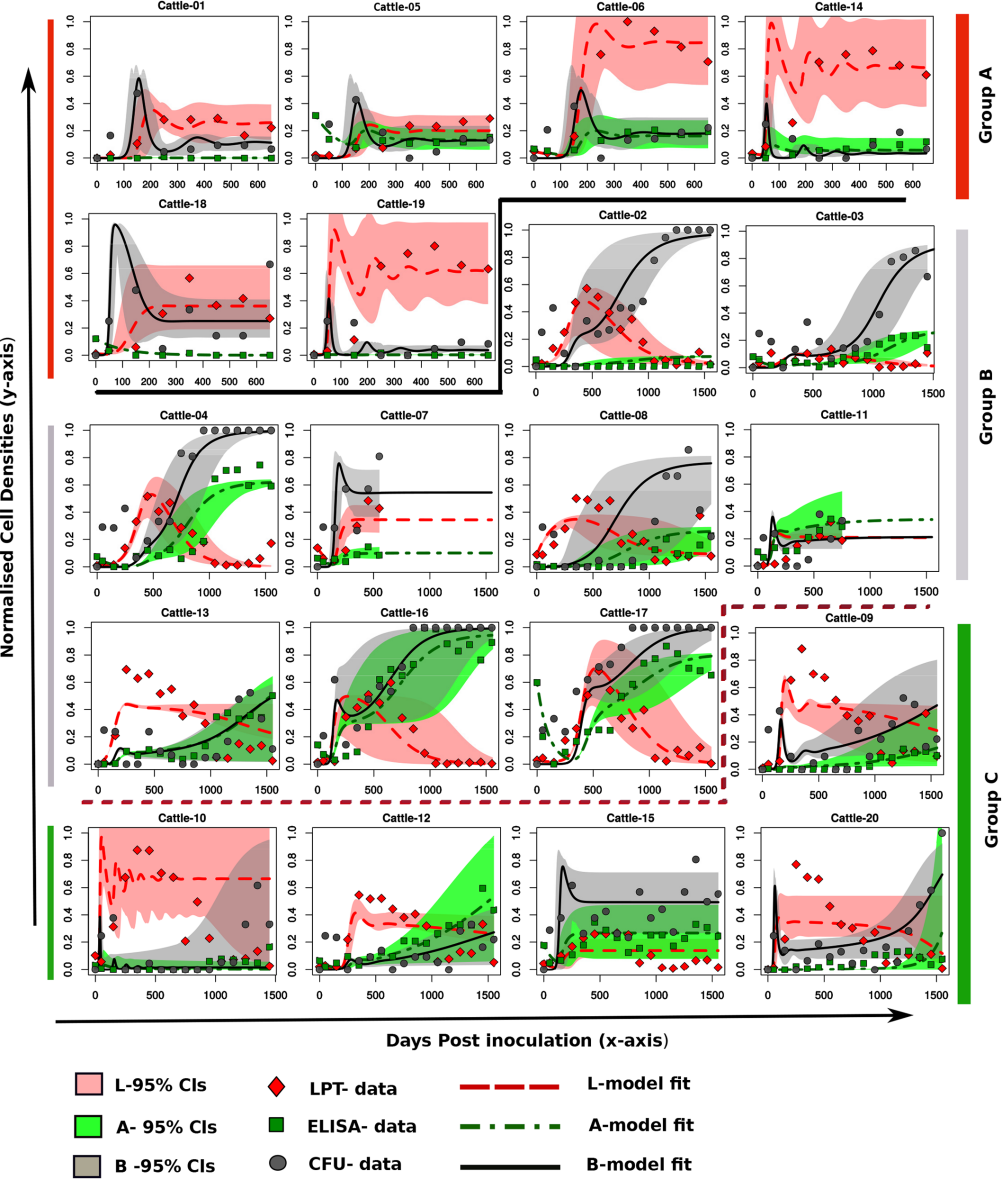
Comparing Models to Cattle MAP Experimental Infection Data. Through fitting the model to cattle data, all animals can be categorized into three distinct groups that correspond to three different models that predict unique immune response interactions with MAP bacteria. Group A is a set of animals with data that can be reproduced with Model A. Data for cattle in Groups B and C can be explained with Models B and C, respectively. Interactions and model equations that describe Models A, B, and C are given in Fig 3. The predicted immune interactions are different between these groups; however, within each group, different immune responses are explained by similar mechanisms with different estimated parameter values. Lines show model predicted trends, while shapes (circle, square, diamond) represent experimental data (CFU, ELISA, LPT, respectively). The red line represents the model-simulated LPT/CMI response, the green line represents the AMI response, and the black line stands for CFU kinetics.

In model selection, the least squares estimates (LSE), which is basically equal to a Gaussian maximum likelihood estimate (MLE), was used. Once cattle were assigned to their respective groups, we used the Markov Chain Monte Carle (MCMC) method based on a Bayesian framework implemented in the FME package in R [28]. We therefore used a Gaussian likelihood to draw model parameter posteriors assuming uniform non-informative priors while the variances were regarded as nuisance parameters. The MCMC chain was generated with at least 50,000 runs for the final fitting of each animal. Chain convergence was examined visually, and extended runs were carried out in cases in which convergence was not evident. Uncertainty of each estimated parameter was evaluated by analyzing the MCMC chains by calculating the 2.5 and 97.5 quantiles of the chain around its median to give the 95% credible intervals (CIs) (see model fits in Fig 2 and estimated parameters in Table 1).

### Multivariate linear regression analysis

Mathematical models were used to place animals in different groups by fitting the models to data for each animal. These models predicted the mechanistic interactions between the measured biological values for each animal. However, we further used multivariate linear regression to ascertain the predicted relationships in a more simple way. The general belief is that LPT (CMI) responses are protective; therefore, they should be negatively correlated with CFU shedding. On the other hand, AMI responses should be positively correlated with CFUs since their level of expression generally increases with CFU shedding and disease progression. The regression model *B*_*tot*_ = *β*_0_ + *β*_1_*L* + *β*_2_*A* + *error* is used to predict the relationship among measured CFUs (*B*_*tot*_) shed in the feces, and the expressed immune responses LPT (*L*) and AMI (*A*) in course of MAP infection/disease progression. The parameters *β*_*0*_, *β*_*1*_, and *β*_*2*_ are the relationship predictors and are estimated by fitting the regression model to the clustered data for each group of animals, and *error* is the measure of the deviation between the observed data and the fitted model. A positive or negative value of a predictor parameter implies a positive or a negative correlation. In the case that *β*_*0*_ is predicted to significantly correlated with CFU shedding while *β*_*1*_ and *β*_*2*_ are not, it is then interpreted that the observed relationship is from a signal that is not related to the role of the expressed immune responses.

### Model simulation results and uncertainty analysis

The uncertainty in model output variability is evaluated by carrying out multivariate parameter sensitivity analysis using Latin hyper cube sampling (LHS) and then evaluating how the variation in model parameters affect the variability of the model-simulated trajectories using the FME package in R [28]. The uncertainty in the model-simulated results is quantified by calculating quantiles of the output trajectories or through enumerating the mean of the trajectories and evaluating how the rest of the results deviate from the mean trajectory. Further variation in the model-simulated results is also evaluated by comparing the mean trajectory with the minimum and maximum trajectories that correspond to the entire sampled parameter space.

## Results

### Comparing models with cattle experimental infection data

**Fig 3:**
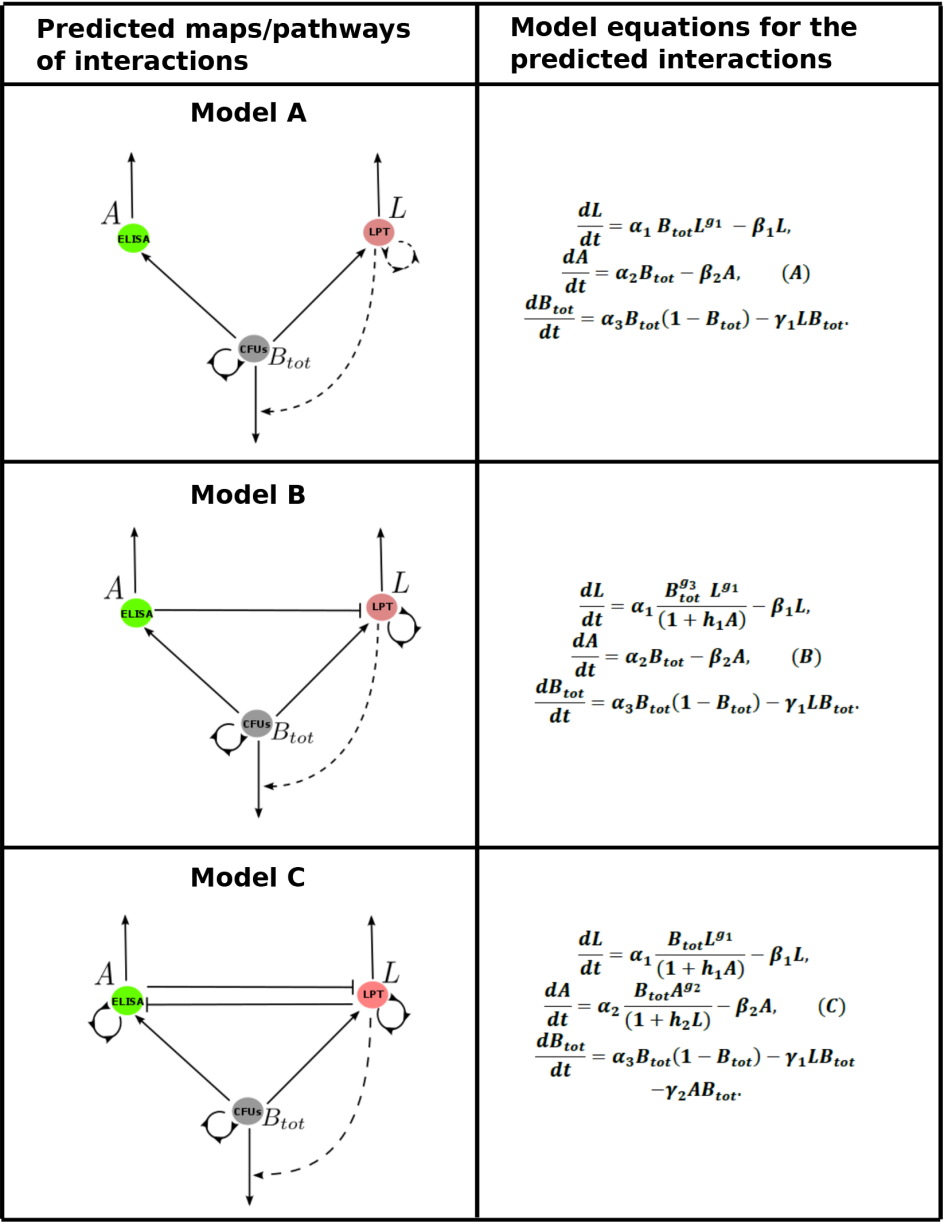
Predicted immune interactions that explain MAP shedding patterns. In graphs for Model A, B and C, lines ending with an arrow indicate stimulation (induction or up-regulation), lines ending with a flat bar represent suppression or inhibition and broken lines represent predicted effects that have varying effects (from strong to week) within each group, while solid lines represent strong effects within a group. Lines represented with a semi-circle with arrows represent self-stimulation/proliferation or auto-regulation.

Applying the algorithm explained in the methods section, we predicted several mathematical models (Models A, B, C, … N, see the model selection algorithm, Fig 1B) shown in the supplementary file. From the predicted models, three models (Fig 3) were selected for simpler structure and better fitting to data (S2 Table).

Fig 2 shows experimental data (LPT, ELISA, and CFU) and fitting of model outputs in 20 animals. As mentioned above, the animals could be separated into three groups. Models A, B, and C (Fig 3) were used to fit experimental data of Groups A (Fig 2 top), B (Fig 2 middle), and C (Fig 2 bottom), respectively. The interaction pathway shown in Fig 3, Model A, suggests that the LPT response is replenished through mechanisms such as proliferation or differentiation of lymphocytes. We observed that a high LPT response matched with low CFU and low expression of AMI. This observation suggests that LPT is protective in this group of animals. Also, both the LPT and AMI responses could be potentially generated in proportion to CFU. The CFU is predicted to induce the development of both LPT and AMI responses, while at the same time, CFU increases through replication. The predicted interactions suggest that the LPT response could be active in eliminating the pathogen (represented by CFU), though at different strengths. The model predicts varying effects of the LPT immune response in pathogen elimination, from being relevant (strongly expressed) to being non-relevant (weakly expressed) between animals. The AMI response is predicted as non-essential in explaining the data, and therefore is predicted to be non-protective in Group A and Group B.

We also observed that in Group B, there was high expression of a non-protective AMI response that was accompanied by high CFUs. Model B suggests that accumulation of MAP bacteria (reflected by CFUs) drives expression of an AMI response which then replaces an initially dominant LPT immune response. Accumulation of CFUs is another distinct facet of Group B animals, which is accompanied by increased AMI and low LPT response. This pattern can be clearly explained by looking at the predicted immune pathway for this group of animals (pathway/map B or Model B). Model B suggests that the AMI response suppresses the LPT response, hence predicting that accumulation of CFUs (which should stimulate both responses) will favor AMI expression, since AMI is predicted to suppress the LPT response. The strength for the LPT response corresponds to the level of the excreted MAP bacteria (CFUs) and varies between animals. High expression of LPT response confers protection; hence, low CFUs are observed in Group A. Low expression of LPT response is accompanied by high CFUs and high expression of AMI responses. Group B shows that 6/9 (66.66%) animals in this category exhibit an initial high expression of the LPT response and then a switch to dominance of AMI (also see S2 Fig, for cattle 7 and 11 fits without projections going up to 55 months). This switch is observed to occur as CFUs increase. In our previous study [6], we used a mathematical model to show that free bacteria favors selection for Type 2 (AMI) immune cells, which are not protective. On the other hand, the development of CMI cells (measured by LPT expression) will suppress bacterial growth, hence the low observed CFUs. In Group C, cross inhibition between LPT and AMI responses is predicted. However, LPT response is protective, and AMI response is not protective. The LPT response protection strength is predicted to vary between animals such high expression of LPT is matched with low bacterial shedding, and low LPT expression is accompanied by high CFUs (high bacteria shedding). CMI and AMI cross suppression, and AMI proliferation are the key immune mechanisms that differentiate Group C from Groups A and B.

### Predicted immune response and MAP bacteria interaction pathways

Fig 3, Model A presents the simplest predicted interaction pathway between the immune responses (CMI and AMI) and MAP bacteria (CFU). Both CMI and AMI are stimulated by bacterial load (CFUs), and no interaction (cross inhibition or suppression) was predicted between the two immune responses. The infection/immune response pathway (Fig 3, Model B) has some similarity to Model A; however, it has one additional unique predicted mechanism: unlike in Model A, Model B suggests that LPT responses are suppressed by AMI, and this inhibition is an important mechanism to explain data in this group. Also, AMI responses are predicted to replenish. Elimination of MAP bacteria (CFUs) by LPT responses is predicted to be important in all the animals in Group B, like animals in Group A. Model C suggests that both LPT and AMI responses will self-replenish. In this model, AMI and LPT can cross suppress, both expanding with increasing bacterial burden and proliferating to sustain their respective populations even though they are antagonistic.

### Revealing the differences between the predicted animal groups using linear regression

**Fig 4:**
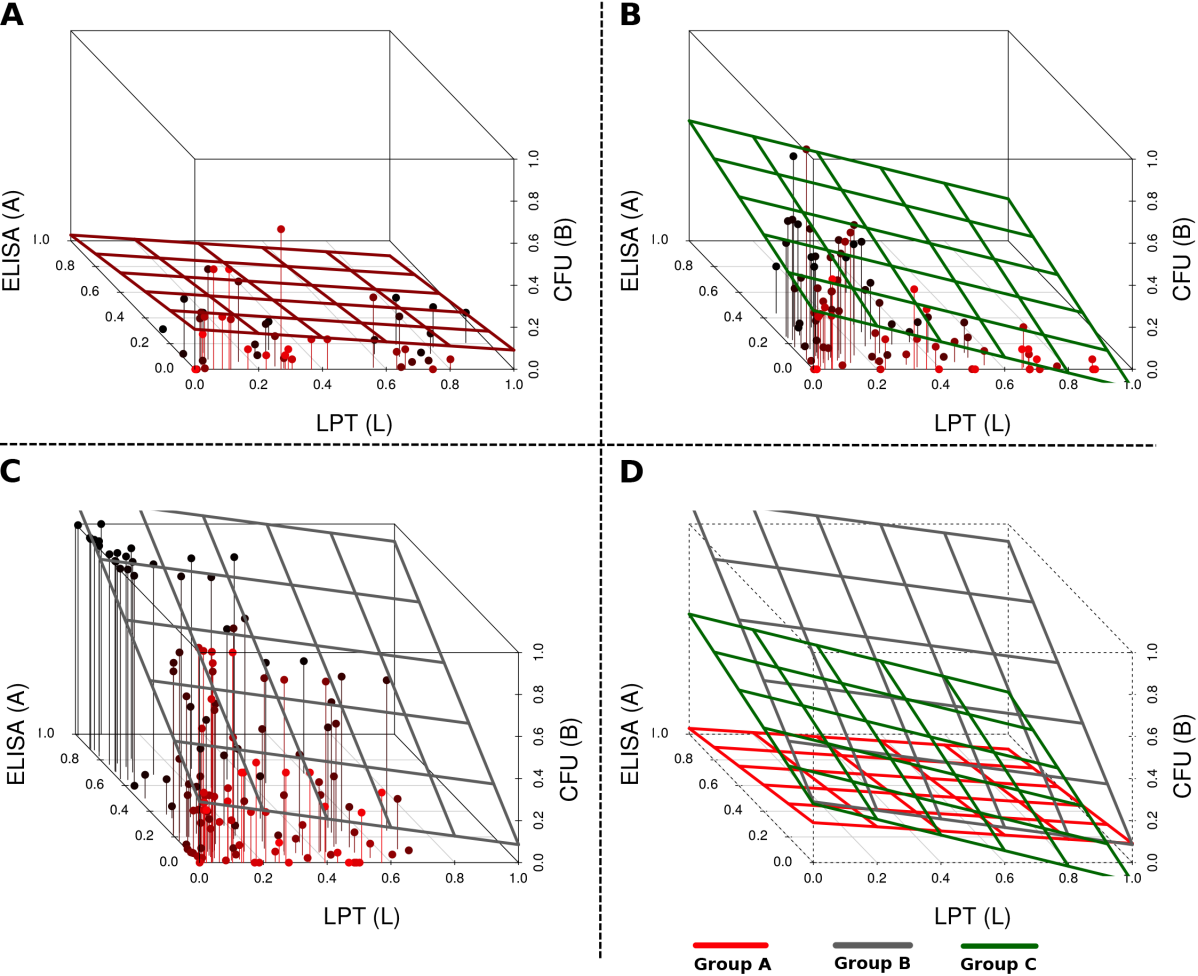
Data regression analysis. A multivariate linear regression analysis for B (CFUs), CMI (LPT) and antibody (ELISA) (*B*_*tot*_ = *β*_0_ + *β*_1_*L* + *β*_2_*A* + *error*). CFUs (*B*_*tot*_) are used as the dependent variable and CMI (*L*) and AMI (*A*) responses as independent variables. The scatter plots, Panel A (Group A), Panel B (Group B), and Panel C (Group C) illustrate the differences between the 3 predicted models that are shown in Fig 3 to explain data for all the animals. Through fitting a plane, the scatters clearly makes the difference between the three groups vivid. The fitted plane for Group A shows a relatively low LPT expression to be correlated with low antibodies and low MAP shedding. The Group B fitted plane shows high CFU shedding to be correlated with low LPT expression (high LPT is associated with low MAP CFU counts and antibody expression). The plane for Group C suggests MAP shedding to be associated with low LPT expression and low levels of antibodies. Panel D, illustrates how the fitted planes for all the groups are different and distinct.

In Fig 3, AMI and LPT response pathway interaction with MAP bacteria are shown pictorially, and the corresponding system of equations (A, B, C) that model the interactions in each pathway are given. The pathways show maps of the immune mechanisms that are predicted to be stimulated, suppressed, or unchanged between animals. The corresponding estimated parameters for these mechanisms are given in Table 1 and they show the varying magnitudes/influence of these immune mechanisms between animals. In Fig 4, we used a multivariate linear regression model (*B*_*tot*_ = *β*_0_ + *β*_1_*L* + *β*_2_*A* + *error*) to predict if AMI and LPT responses correlate with CFUs and further illustrate the distinct differences between the three predicted groups. The Group A fitted plane’s intercept is predicted to be significant, while LPT and ELISA (AMI) are negatively related to CFU shedding; however, this relationship (*R*^*2*^ = 0.06 and *R*^*2*^ adjusted = 0.01) is not significant (*p* = 0.28), and the predicted relationship is CFU = 0.19–0.10L–0.16A. In Group B, the plane’s intercept is predicted to be significant, while LPT is negatively related (*p* = 0.12) and ELISA (AMI) is positively related (*p* < 2.23E-16) to CFU shedding. In this group, regression analysis predicts ELISA (AMI) response to increase with CFU shedding while LPT decreases with increasing (or vice-versa) CFU shedding (CFU = 0.29–0.20 L + 0.83A, *p* = 2.2E-16, *R*^*2*^ = 0.43, adjusted *R*^*2*^ = 0.42). In Group C, CFUs were predicted to be significantly negatively correlated with LPT response (*p* = 6.21E-5), regardless of how positively correlated with the ELISA (AMI) response (*p* = 0.11), as reflected in the predicted relationship CFU = 0.28–0.37 L + 0.29A (*p* = 5.05E-6, *R*^*2*^ = 0.26, adjusted *R*^*2*^ = 0.24). In general, the AMI response is predicted to be positively related with CFU shedding while LPT response is negatively related to CFUs (Groups B and C). However, in Group A, both the AMI and LPT responses are negatively related to CFUs. These predicted relationships agree with the predictions of the dynamic models, which show AMI responses to increase with CFUs, while increasing LPT response is followed by suppressed MAP bacterial growth (reduced bacteria shedding). Fig 4D shows that the fitted planes are different. Though multivariate regression analysis cannot explain the time kinetics of the data, it does, however, show how different these groups are.

### Uncertainty in predicted CMI, AMI, and CFU trajectories to model parameters

**Fig 5:**
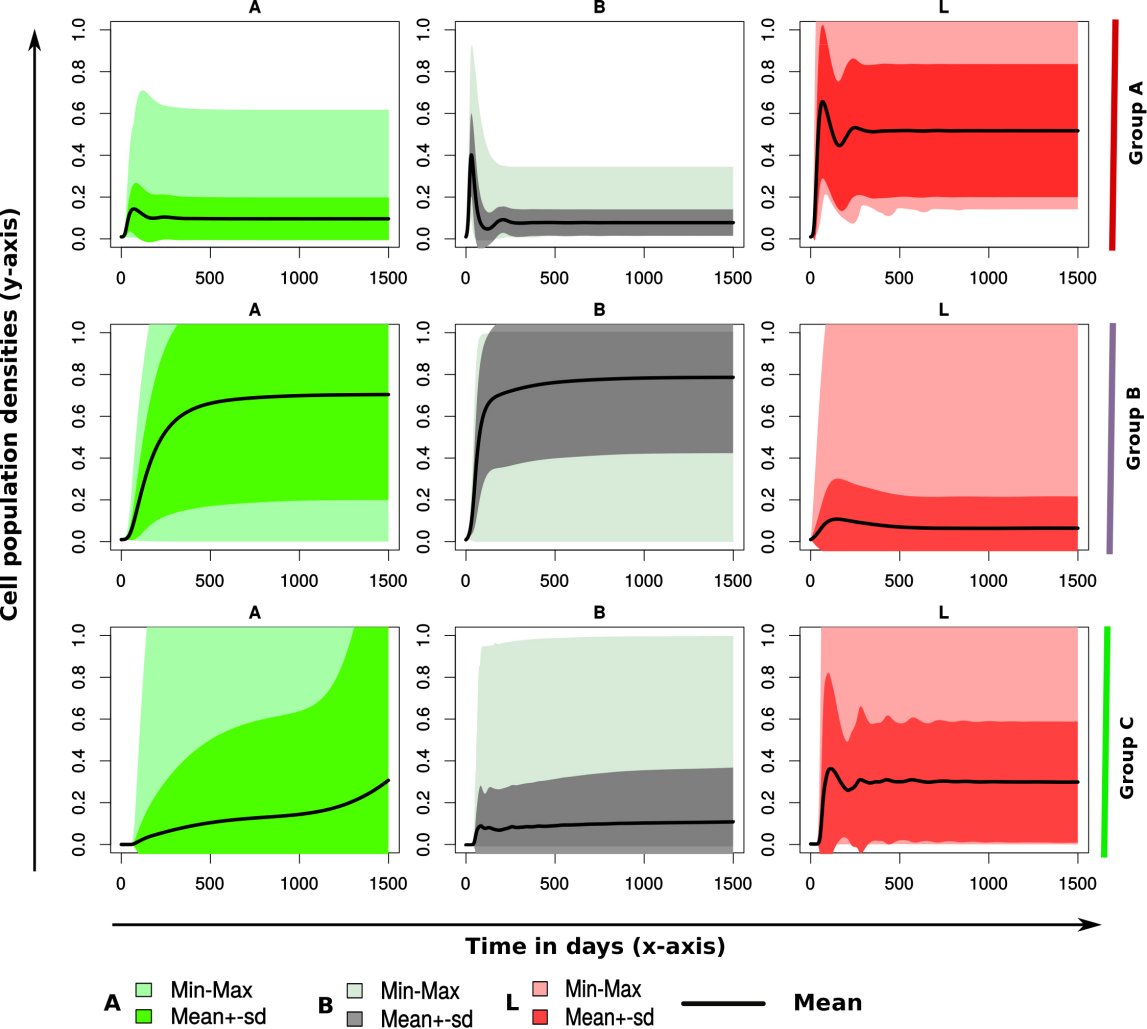
Group Summary Dynamics. The upper row shows trajectories of CFU shedding and immune response variables using summary parameters for Group A. The middle and lower rows show corresponding summary dynamics for Groups B and C, respectively. Summary parameter statistics for each parameter were derived by finding the average of each parameter in each group, and the uncertainty in the trajectories were generated by carrying out multivariate sensitivity analysis (using LHS method), hence generating the shaded regions, where min and max correspond to the minimum and maximum group trajectories generated and the solid line represents the group mean.

Fig 5 show simulated summary group dynamics. Both CFU and immune response trajectories are simulated using summary parameter estimates for each parameter within each group. Summary parameter estimates were obtained by averaging similar parameters in each group (for example, a summary parameter for *α*_*1*_ in Group A is calculated by summing all *α*_1’_s in this group and then dividing by the number of animals within the group), or by calculating the median of the parameter (see S3 Fig). Uncertainty of the model output variability is then evaluated by mapping several trajectories by multivariate parameter variations between the minimum and the maximum parameter values using the LHS method, as shown by the shaded regions in Fig 5 (or using the quantiles around the median as shown in S3 Fig). The summarized dynamics demonstrate the differences between the CFU patterns and the immune response variables in each group and illustrate the general differences in disease progression kinetics between the groups.

## Discussion

There are variant disease outcomes following cattle exposure to MAP. However, not all animals that are exposed subsequently get infected and of those that do, disease pathology is not homogeneous. Heterogeneous infection pathology is evidenced in the intermittent and unpredictable shedding patterns observed in individual infected cattle (See S3 Table, for animal summary demographics and immune response peak times and values). Therefore, deciphering the host immune response mechanisms associated with the different bacteria shedding patterns is a complex and a daunting task. It can be speculated that the variation of the time to shedding could be correlated to differential, complex coordination of immune response dynamics as the host transitions through the different stages of infection and disease.

In this study, we used an inverse modeling approach that assumed no prior knowledge of the immune pathway that is involved in controlling the way the infected animals shed MAP in feces. We used this approach to predict the essential immune mechanisms from a pool of several possible mechanisms by identifying and returning the essential minimal interactions that can reproduce the trends in the data. We predicted and explained combinations of potential cattle immune response mechanisms that can explain the fecal shedding patterns observed in the infected cattle and how they differ between the predicted different groups. Furthermore, basic statistical analysis of the experimental data using multivariate linear regression models revealed that the three predicted animal groups are statistically different through fitting a plain for each category (Fig 4).

We tested the developed models with immune response (LPT and AMI) data and MAP shedding data (*B* or *B*_*tot*_ [CFU counts of MAP bacteria in feces]) that were obtained from 20 cattle experimentally infected with MAP and followed for 55 months. The modeling approach predicted that the main differences between the groups were the magnitude of the common fitted parameters and the parameters that are not common between the different groups. However, results from Fig 2 and Fig 3A, 3B and 3C show that even though these groups generate distinct patterns, considering multivariate sensitivity analysis, that span through the entire space of the estimated parameters generate a wide range of different infection/disease trajectories (Fig 5). It is imperative to note that variations between these parameters can generate several different MAP shedding and immune response kinetics.

It is generally accepted that the ability of mycobacterial pathogens like *Mycobacterium tuberculosis* and MAP to replicate and persist in macrophages is a central mechanism for their pathogenesis. In fact, MAP has been shown in several studies [29, 30] to exhibit different intracellular rates of replication, avoid killing by macrophages, and to have the ability to establish prolonged residence in macrophages. Different rates of differentiation for Th1 and Th2 cells can skew immune response selection along a specific pathway/lineage and hence result in different Th1/Th2 response dynamics (reviewed in [31]). In MAP infection, the dominance of the antibody response is associated with multi-bacillary lesions and rapid disease progression. The differing ability of bovine monocyte cell lines to ingest and restrain intracellular growth of MAP potentially contributes to the observed heterogeneity in lesion patterns and disease progression, and hence, different shedding kinetics. Accumulation of bacteria can lead to the differential selection of Th2 cells and sustained stimulation of the B cell antibody response.

Applying the inverse modeling approach to analyze the data, we predicted three different ways LPT and AMI potentially interact with the MAP pathogen. These three different pathways reasonably explain data for a subset of the 20 cattle. We predicted that there are unique immune response mechanisms that are different between the three predicted immune/pathogen interactions. Model fitting to data made it possible to discriminate those immune mechanisms that were not expressed or suppressed from the expressed mechanisms and to predict how the expressed immune response mechanisms correlate with bacterial shedding patterns. These results give insight into the speculated theory that the observed immune response is a reflection of bacterial load and the ongoing battle between the immune response and the infection. Also, considering that all animals predicted to be in Group A died before the end of the experiment, there is a possibility that animal grouping prediction outcomes could have been different if a similar time period equal to the time period of animals in Group A was used. However, the model/grouping selection criterion used was based on observed expressed immune responses and CFUs excreted during the entire period of the experiment or as long as the animal was alive. That is, the observed data for animals predicted to fall into Group A enable the models to predict immune mechanisms that were expressed before their death. Unlike in the other groups, data beyond the lifespan of animals in Group A include more information (information not observed in Group A) to explain the long-term effects of MAP on immune responses and CFU shedding kinetics. Furthermore, the long-time dynamics of Group A were simulated using summary parameters for this group to generate CFU kinetics and immune variable trajectories that are different from those of Groups B and C (Fig 5).

Our analysis reveals that the CMI and AMI responses expressed by animals in Group A are more of an indication of the presence of infection. High to medium expression of CMI is predicted to expand as a result of pathogen stimulation and through proliferation, and is responsible for the suppressed bacterial growth that is observed in animals in this group. The low CFU bacterial shedding that followed was commensurate with minimal AMI response (predicted to expand only as a result of pathogen stimulation or proportional to the bacterial density) that was expressed. This observation supports the current belief that CMI responses are protective, while AMI (which is not protective) expression increases with disease progression, as predicted using the regression models (Fig 4) that AMI responses increase with CFU shedding. In Group B, AMI responses were predicted to suppress CMI responses; therefore, we observed increased CFU shedding as CMI expression plummeted. We could clearly see the early expressed CMI response getting replaced with the AMI response. Increased bacterial burden (CFU shedding) was expected in this group, because once the CMI response is lost, bacterial growth will be unrestricted, since our model (Model B) predicts a non-protective role for the AMI responses. Also, the AMI response was expected to expand (as seen in Fig 2, Group B) since its expression was stimulated directly by the MAP pathogen. As a result, we saw increasing growth trajectories for CFUs and AMI populations for most of the animals in Group B, Fig 2. Immune responses and bacteria biological interactions for animals in Group C were more complicated. No distinct patterns clearly stood out; however, it was visible that CFU shedding increased over time, but not as distinctively as in Group B. Also, we observed AMI expression increasing concomitantly with CFU shedding. This observation was consistent with observations in Groups A and B, as well as with the regression correlations predicted in Fig 4. However, other patterns suggest intermediate expressions for both CMI and AMI responses, and the magnitudes of these expressions vary between animals and are matched with different CFU shedding kinetics. These observations demonstrate that the variation of the time to shedding and the variable expression of CMI/AMI responses reflect the ongoing intricate pathogen-immune response interactions within the host as individual animals transition through the different stages of the infection. Also, in Group C, Model C did not adequately explain the LPT kinetics, which seemed to suggest that there could be a mechanism that could not be captured by even the most complicated model. However, the model is still able to provide a picture of the probable LPT dynamics. Our models demonstrate that animals do share some similar immune response mechanisms, but these similarities could be stimulated or expressed disparately in each animals (see Fig 3), hence the myriad of immune response kinetics and CFU patterns that were evident in each animal and in each group (Fig 2 and Fig 5). This result also explains why some animals could be non-progressors or slow-progressors and others could be fast progressors [7]. Also, the shedding patterns that follow the differentially expressed immune responses in part predict and explain the biology of low shedders and high shedders. High CMI response predicts low shedding, high AMI predicts high shedding, and combined AMI and CMI responses can explain different shedding patterns that range from low to high but predominantly intermediate shedding (see Figs 2 and 5 and S3 Fig).

There is shortage of data that describe the relationship between MAP CFU counts in intestinal microbiota flora and MAP bacteria density in tissue/lesions, or data that correlates excreted MAP to MAP density at the site of infection. Also, the mechanisms as to how MAP bacteria exit from tissue lesions, infected macrophages, and Peyer’s patches into the gut for excretion are not clearly understood. Our models are limited in dealing with these biological realities; therefore, the availability of experimental data and biological insights underlying these mechanisms will be beneficial in not only refining the models presented in this study but also in general in the area of theoretical modeling to assist with providing predictions, explanations, and insights to address the pressing questions in this field. Another shortfall of our models is that they fail to explain in detail the erratic fluctuations of MAP in feces. Possibly this is contributed by the modeling approach that made use of deterministic differential equations and the data smoothing done before fitting the models to the data. This could also be related to sampling strategy and poor sensitivity of the fecal culture method. However, in general, the model fitting gives a clear picture of how immune responses were different between individual animals through different magnitudes of fitted parameters and between groups of animals through fitted different immune mechanisms. Also of importance is the ability of the models to identify immune mechanisms that are engaged/active and common between different animals and those that are expressed while suppressed (not stimulated) in other animals. There are similar predicted immune mechanisms between all animals in the different groups. Also, a clear picture of the differences between these groups is illustrated (see Models [maps/pathways] A, B, and C in Fig 3 and fitted plains for scatter plots in Fig 4.

In conclusion, our mathematical models show that the MAP shedding patterns observed in the progression of MAP infection can be explained by the expressed immune responses. In general, our results indicate that bacterial load is correlated with the immune response that will be predominantly expressed. High bacterial burden is accompanied by low expression of AMI, and a high LPT response is matched with low bacterial load. When high bacterial load is observed, it could be a result of the predominant expression of AMI that fails to control bacterial growth and immune response selection pressure that favors the AMI response that in turn replaces the LPT response.

## Supporting Information

S1 Fig Potential Biological Interactions. This map shows potential interactions between the LPT/CMI cell response, ELISA (humoral response/AMI), and CFUs (MAP bacterial density) at the site of infection. Interactions represented here assume that CMI and the antibody/humoral response cross suppress, that free bacteria will drive development of both immune responses (CMI and AMI), and that the immune responses reduce the MAP population.
(PDF)

S2 Fig Fits for Cattle 7 and 11. Cattle 7 and 11 were predicted to be in Group B even though they had a shorter time span than the rest of the animals within this group.
(PDF)

S3 Fig Group Summary Dynamics. Group dynamics for CFU shedding and immune response variables. Panels A, B and C show the summary dynamics for Groups A, B and C, respectively. Shaded regions represent the 5^th^ to 95^th^ quantiles around the model median (that correspond to the summary statistic parameters which is the group median). This is in contrast to the group mean that was used in Fig 5.
(PDF)

S1 Table Calculated AIC Values. Model AIC computed values for model selection and comparison.
(DOCX)

S2 Table Model Comparisons. Illustration of how model comparison and selection was carried out. We selected Cattle 01 (Group A), 02 (Group B), and 15 (Group C) as examples to demonstrate the entire model selection process. Models with a simpler structure and fewer terms (less complicated) were given precedence over complicated models as long as they could explain the data (a smaller RSS and AIC). For Cattle 01, Model A has a similar RSS compared to Model B and Model C, but with a relatively less AIC and a simpler model structure. Model B is best to explain Cattle 02, while Cattle 03 is best explained by Model C.
(DOCX)

S3 Table Summary of data peak times and values and times and reasons cattle were culled. (DOCX)

S1 Text Predicted models and model parameter identifiability. S1 Text gives a list of potential models that were tested using the model selection algorithm. Final computed AIC values for each animal are given (S1 Table) and an illustration of model (models A, B and C) comparison using a few selected animals in different groups is presented (S2 Table) and an example that demonstrates how model parameter identifiability was carried out. The list of models presented in S1 Text is not exhaustive, it is meant to illustrate the iterative selection process starting with a complex model (Model N) until Model **A**. In our model comparison, the model labelled Model **A,** which is the simplest model could not explain data for any of the infected animals and Model **B** was selected as the best model for Group **A** animals. To illustrate the selection, note that here we have models **A**, **B**, **C**, and **D** that seem to have a similar structure but with different complex interaction terms. Model **D** can explain data for Group **A** animals but this is also true for models **B** and **C**, but Model **B** will be selected because is it simpler. However, Model **A** can explain some of the animals but not all, therefore again Model **B** is selected, even though it is a bit more complicated than Model **A**.
(DOCX)
