# Supplementary figures and images for: Can Immune Response Mechanisms Explain the Fecal Shedding Patterns of Cattle Infected with Mycobacterium avium Subspecies paratuberculosis?
Source: PLoS One. 2016 Jan 25;11(1):e0146844. doi: 10.1371/journal.pone.0146844 (PMC4725749; doi:10.1371/journal.pone.0146844)

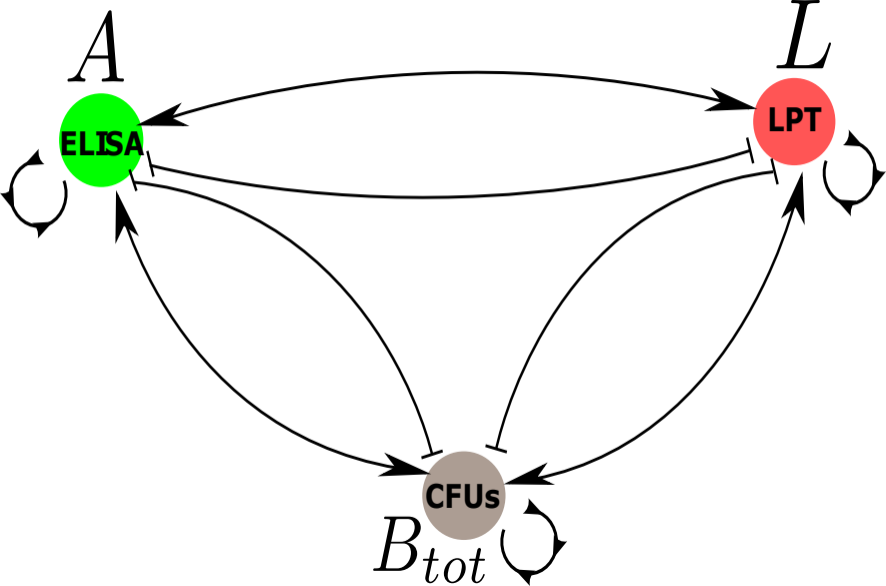

Supplement: S1 Fig — This map shows potential interactions between the LPT/CMI cell response, ELISA (humoral response/AMI), and CFUs (MAP bacterial density) at the site of infection. Interactions represented here assume that CMI and the antibody/humoral response cross suppress, that free bacteria will drive development of both immune responses (CMI and AMI), and that the immune responses reduce the MAP population. (PDF) [file pone.0146844.s001.pdf]

Cattle-07

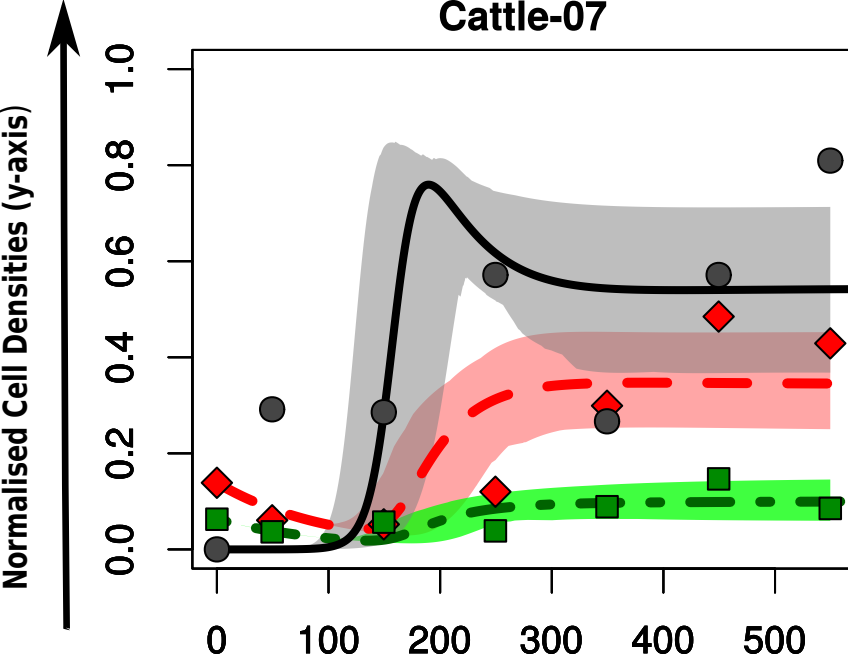

Cattle-11

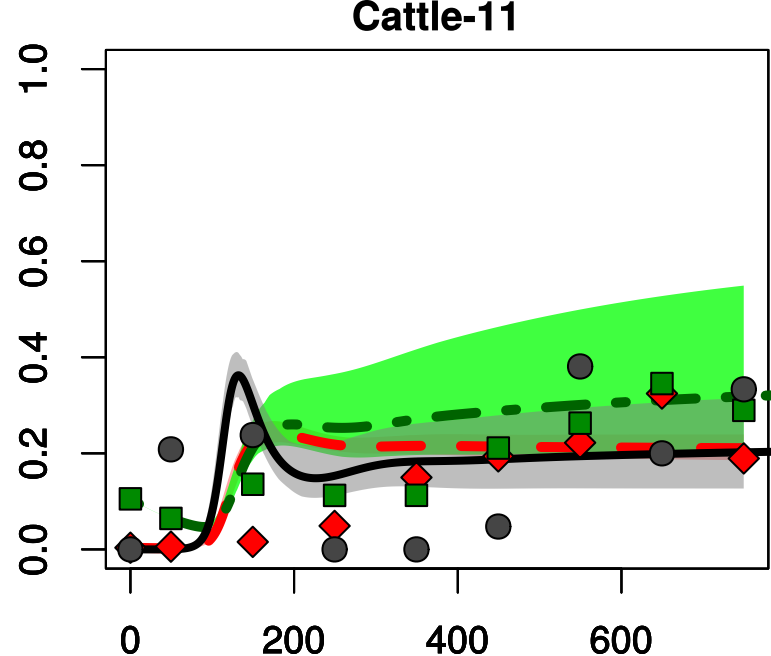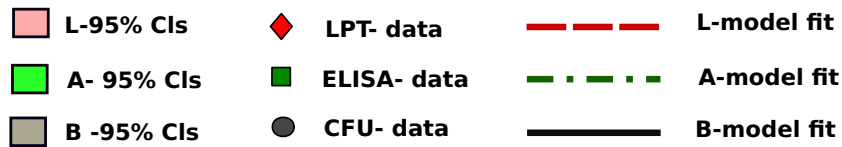

Supplement: S2 Fig — Cattle 7 and 11 were predicted to be in Group B even though they had a shorter time span than the rest of the animals within this group. (PDF) [file pone.0146844.s002.pdf]

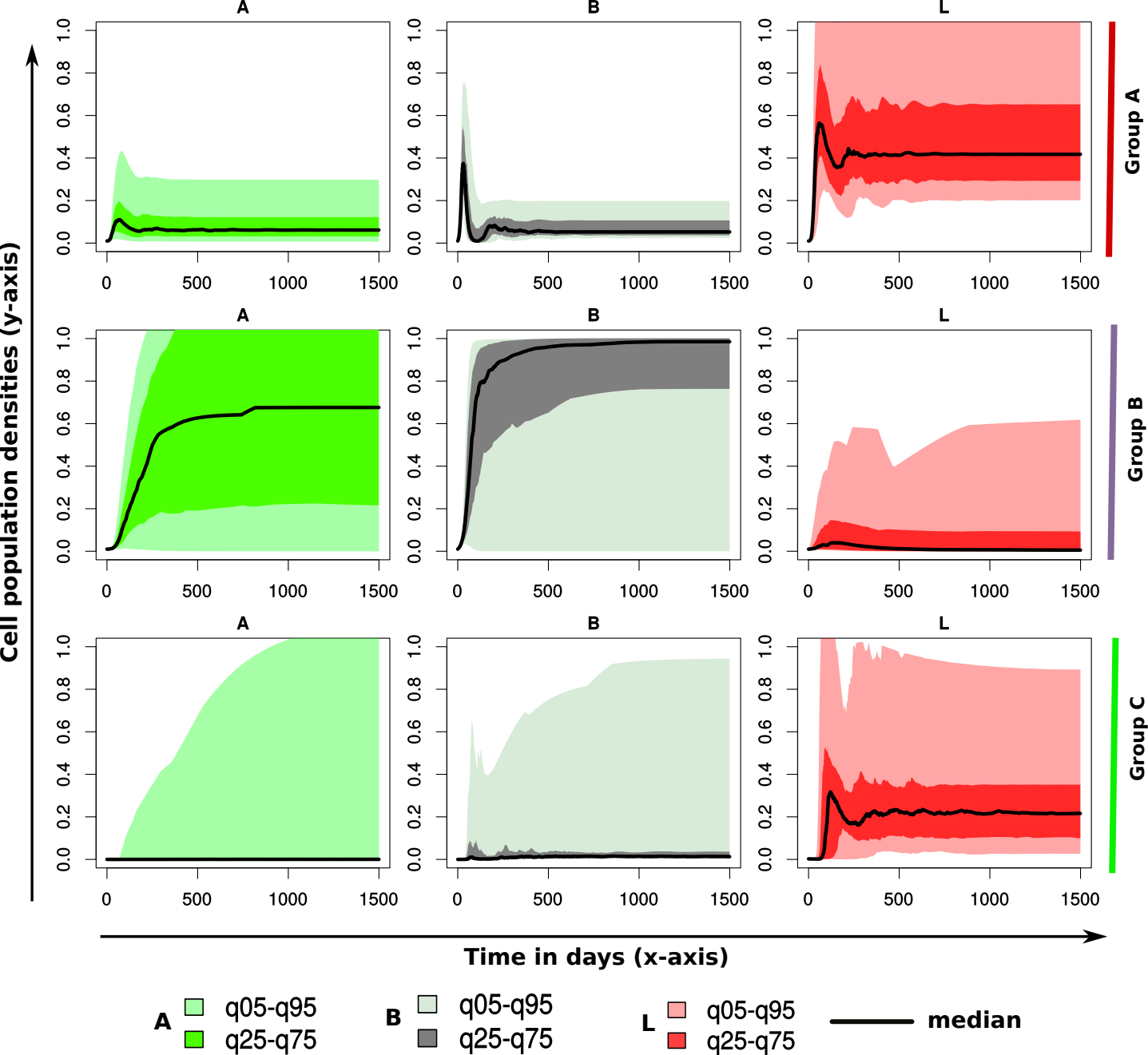

Supplement: S3 Fig — Group dynamics for CFU shedding and immune response variables. Panels A, B and C show the summary dynamics for Groups A, B and C, respectively. Shaded regions represent the 5th to 95th quantiles around the model median (that correspond to the summary statistic parameters which is the group median). This is in contrast to the group mean that was used in Fig 5. (PDF) [file pone.0146844.s003.pdf]
