# Supplementary material for: Can Immune Response Mechanisms Explain the Fecal Shedding Patterns of Cattle Infected with Mycobacterium avium Subspecies paratuberculosis?
Source: PLoS One. 2016 Jan 25;11(1):e0146844. doi: 10.1371/journal.pone.0146844 (PMC4725749; doi:10.1371/journal.pone.0146844)
Supplement: S1 Table — Model AIC computed values for model selection and comparison. (DOCX) [file pone.0146844.s004.docx]

**S1 Table.** Calculated AIC values

| **Cattle** | ***k*** | **RSS** | **n** | **AIC** |
| --- | --- | --- | --- | --- |
|  |  |  |  |  |
| **C01** | 5 | 0.04 | 24 | -56.67 |
| **C05** | 5 | 0.14 | 24 | -43.61 |
| **C06** | 5 | 0.17 | 24 | -41.59 |
| **C14** | 5 | 0.14 | 24 | -43.61 |
| **C18** | 5 | 0.34 | 24 | -34.37 |
| **C19** | 5 | 0.25 | 24 | -37.57 |
|  |  |  |  |  |
| **C02** | 6 | 0.45 | 51 | -92.77 |
| **C03** | 6 | 0.35 | 51 | -90.58 |
| **C04** | 6 | 0.77 | 51 | -80.87 |
| **C07** | 6 | 0.30 | 21 | -26.74 |
| **C08** | 6 | 0.88 | 51 | -77.91 |
| **C11** | 6 | 0.21 | 27 | -44.94 |
| **C13** | 6 | 1.05 | 51 | -74.01 |
| **C16** | 6 | 0.86 | 51 | -78.42 |
| **C17** | 6 | 0.65 | 51 | -84.62 |
|  |  |  |  |  |
| **C10** | 7 | 0.80 | 48 | -71.35 |
| **C09** | 7 | 1.05 | 48 | -72.01 |
| **C12** | 7 | 0.62 | 51 | -83.67 |
| **C15** | 7 | 0.54 | 51 | -86.73 |
| **C20** | 7 | 0.81 | 51 | -77.75 |
