# Supplementary material for: Can Immune Response Mechanisms Explain the Fecal Shedding Patterns of Cattle Infected with Mycobacterium avium Subspecies paratuberculosis?
Source: PLoS One. 2016 Jan 25;11(1):e0146844. doi: 10.1371/journal.pone.0146844 (PMC4725749; doi:10.1371/journal.pone.0146844)
Supplement: S2 Table — Illustration of how model comparison and selection was carried out. We selected Cattle 01 (Group A), 02 (Group B), and 15 (Group C) as examples to demonstrate the entire model selection process. Models with a simpler structure and fewer terms (less complicated) were given precedence over complicated models as long as they could explain the data (a smaller RSS and AIC). For Cattle 01, Model A has a similar RSS compared to Model B and Model C, but with a relatively less AIC and a simpler model structure. Model B is best to explain Cattle 02, while Cattle 03 is best explained by Model C. (DOCX) [file pone.0146844.s005.docx]

**S2 Table: Model comparisons** (Models A, B and C are from the manuscript and Models B, F and I in the supplementary file (S1 Text))**.**

| Animal | **Model A** |  | **Model B** |  | **Model C** |  |
| --- | --- | --- | --- | --- | --- | --- |
|  | RSS | AIC | RSS | AIC | RSS | AIC |
| C01 | 0.04 | -56.67 | 0.04 | -54.67 | 0.04 | -50.67 |
| C02 | 2.35 | -56.06 | 0.04 | -92.77 | 2.35 | -56.16 |
| C15 | 0.71 | -78.67 | 0.04 | -83.53 | 0.54 | -86.73 |
