# Supplementary material for: Can Immune Response Mechanisms Explain the Fecal Shedding Patterns of Cattle Infected with Mycobacterium avium Subspecies paratuberculosis?
Source: PLoS One. 2016 Jan 25;11(1):e0146844. doi: 10.1371/journal.pone.0146844 (PMC4725749; doi:10.1371/journal.pone.0146844)
Supplement: S3 Table — (DOCX) [file pone.0146844.s006.docx]

**S3 Table:** Summary of data peak times and values and times and reasons cattle were culled.

| Groups | Time of Death | Reason of Death | Peak value (normalised) | | | Peak Time (number of days) | | |
| --- | --- | --- | --- | --- | --- | --- | --- | --- |
|  |  |  | LPT | AMI | CFU | LPT | AMI | CFU |
| Group A |  |  |  |  |  |  |  |  |
| C01 | 650 | Infertility | 0.3 | 0.0 | 0.50 | 250 | 0.0 | 150 |
| C05 | 650 | Infertility | 0.3 | 0.15 | 0.40 | 350 | 250 | 150 |
| C06 | 650 | Infertility | 1.0 | 0.20 | 0.40 | 350 | 350 | 150 |
| C14 | 650 | Infertility | 0.8 | 0.15 | 0.25 | 450 | 450 | 50 |
| C18 | 650 | Infertility | 0.6 | 0.0 | 0.50 | 350 | 00 | 150 |
| C19 | 650 | Infertility | 0.8 | 0.0 | 0.25 | 450 | 00 | 50 |
| Group B |  |  |  |  |  |  |  |  |
| C02 | 1650 | End of Study | 0.60 | 0.0 | 1.00 | 500 | 00 | 1200 |
| C03 | 1650 | End of Study | 0.15 | 0.20 | 0.80 | 700 | 1300 | 1300 |
| C04 | 1650 | End of Study | 0.50 | 0.70 | 1.00 | 500 | 1200 | 800 |
| C07 | 550 | Severe lameness | 0.50 | 0.15 | 0.80 | 450 | 450 | 550 |
| C08 | 1650 | End of Study | 0.50 | 0.20 | 0.80 | 400 | 1200 | 1400 |
| C11 | 750 | Fatty liver syndrome | 0.30 | 0.30 | 0.40 | 700 | 700 | 650 |
| C13 | 1550 | Lameness and mastitis | 0.70 | 0.50 | 0.50 | 200 | 1550 | 1300 |
| C16 | 1550 | Lameness and mastitis | 0.60 | 0.85 | 1.00 | 650 | 1200 | 800 |
| C17 | 1550 | Lameness and mastitis | 0.70 | 0.85 | 1.00 | 550 | 1200 | 800 |
| Group C |  |  |  |  |  |  |  |  |
| C09 | 1650 | End of Study | 0.90 | 0.15 | 0.50 | 400 | 1200 | 1200 |
| C10 | 1650 | End of Study | 0.90 | 0.20 | 0.60 | 400 | 1500 | 1350 |
| C12 | 1650 | End of Study | 0.60 | 0.60 | 0.40 | 350 | 1450 | 1400 |
| C15 | 1550 | Lameness and mastitis | 0.25 | 0.35 | 0.80 | 450 | 450 | 1400 |
| C20 | 1550 | Lameness and mastitis | 0.80 | 0.15 | 1.00 | 250 | 1200 | 1550 |

Peak times are rounded up to the tenth while the peak values are normalized and round to 2 decimal places. To get the actual peak values LPT is multiplied by 10^5.2884, AMI is multiplied by 1.3441 and CFU is multiplied by 3.0. The peak time is represented by the number of days the animal was followed before the peak values were observed.
