# Supplementary material for: Can Immune Response Mechanisms Explain the Fecal Shedding Patterns of Cattle Infected with Mycobacterium avium Subspecies paratuberculosis?
Source: PLoS One. 2016 Jan 25;11(1):e0146844. doi: 10.1371/journal.pone.0146844 (PMC4725749; doi:10.1371/journal.pone.0146844)
Supplement: S1 Text — gives a list of potential models that were tested using the model selection algorithm. Final computed AIC values for each animal are given (S1 Table) and an illustration of model (models A, B and C) comparison using a few selected animals in different groups is presented (S2 Table) and an example that demonstrates how model parameter identifiability was carried out. The list of models presented in S1 Text is not exhaustive, it is meant to illustrate the iterative selection process starting with a complex model (Model N) until Model A. In our model comparison, the model labelled Model A, which is the simplest model could not explain data for any of the infected animals and Model B was selected as the best model for Group A animals. To illustrate the selection, note that here we have models A, B, C, and D that seem to have a similar structure but with different complex interaction terms. Model D can explain data for Group A animals but this is also true for models B and C, but Model B will be selected because is it simpler. However, Model A can explain some of the animals but not all, therefore again Model B is selected, even though it is a bit more complicated than Model A. (DOCX) [file pone.0146844.s007.docx]

**S1 Text. Predicted models and model parameter identifiability**

**Predicted Models**

**Model A**

$$\frac{dL}{dt}=\alpha_{1} B_{tot}-\beta_{1}L,$$

$$\frac{dA}{dt}=\alpha_{2}B_{tot}-\beta_{2}A, (A)$$

$$\frac{dB_{tot}}{dt}=\alpha_{3}B_{tot}\left( 1-B_{tot} \right)-\gamma_{1}LB_{tot}.$$

**Model B** (Model A in the manuscript)

$$\frac{dL}{dt}=\alpha_{1} B_{tot}L^{g_{1}}-\beta_{1}L,$$

$$\frac{dA}{dt}=\alpha_{2}B_{tot}-\beta_{2}A, (B)$$

$$\frac{dB_{tot}}{dt}=\alpha_{3}B_{tot}\left( 1-B_{tot} \right)-\gamma_{1}LB_{tot}.$$

**Model C**

$$\frac{dL}{dt}=\alpha_{1} B_{tot}-\beta_{1}L,$$

$$\frac{dA}{dt}=\alpha_{2}B_{tot}A^{g_{2}}-\beta_{2}A, (C)$$

$$\frac{dB_{tot}}{dt}=\alpha_{3}B_{tot}\left( 1-B_{tot} \right)-\gamma_{1}LB_{tot}.$$

**Model D**

$$\frac{dL}{dt}=\alpha_{1} B_{tot}^{g_{3}}L^{g_{1}} -\beta_{1}L,$$

$$\frac{dA}{dt}=\alpha_{2}B_{tot}-\beta_{2}A, (D)$$

$$\frac{dB_{tot}}{dt}=\alpha_{3}B_{tot}\left( 1-B_{tot} \right)-\gamma_{1}LB_{tot}.$$

**Model E**

$$\frac{dL}{dt}=\alpha_{1} B_{tot}-\beta_{1}L,$$

$$\frac{dA}{dt}=\alpha_{2}B_{tot}^{g_{3}}A^{g_{2}}-\beta_{2}A, (E)$$

$$\frac{dB_{tot}}{dt}=\alpha_{3}B_{tot}\left( 1-B_{tot} \right)-\gamma_{1}LB_{tot}.$$

**Model F** (Model B in the manuscript)

$$\frac{dL}{dt}=\alpha_{1}\frac{B_{tot}^{g_{3}} L^{g_{1}}}{\left( 1+h_{2}A \right)}-\beta_{1}L,$$

$$\frac{dA}{dt}=\alpha_{2}B_{tot}-\beta_{2}A, (F)$$

$$\frac{dB_{tot}}{dt}=\alpha_{3}B_{tot}\left( 1-B_{tot} \right)-\gamma_{1}LB_{tot}.$$

**Model G**

$$\frac{dL}{dt}=\alpha_{1}\frac{B_{tot}^{g_{3}} L^{g_{1}}}{\left( 1+h_{2}A \right)}-\beta_{1}L,$$

$$\frac{dA}{dt}=\alpha_{2}B_{tot}A^{g_{2}}-\beta_{2}A, (G)$$

$$\frac{dB_{tot}}{dt}=\alpha_{3}B_{tot}\left( 1-B_{tot} \right)-\gamma_{1}LB_{tot}.$$

**Model H**

$$\frac{dL}{dt}=\alpha_{1}\frac{B_{tot}^{g_{3}} L^{g_{1}}}{\left( 1+h_{2}A \right)}-\beta_{1}L,$$

$$\frac{dA}{dt}=\alpha_{2}B_{tot}^{g_{3}}A^{g_{2}}-\beta_{2}A, (H)$$

$$\frac{dB_{tot}}{dt}=\alpha_{3}B_{tot}\left( 1-B_{tot} \right)-\gamma_{1}LB_{tot}.$$

**Model I** (Model C in the manuscript)

$$\frac{dL}{dt}=\alpha_{1}\frac{B_{tot}L^{g_{1}}}{(1+h_{2}A)}-\beta_{1}L,$$

$$\frac{dA}{dt}=\alpha_{2}\frac{B_{tot}A^{g_{2}}}{(1+h_{1}L)}-\beta_{2}A, (I)$$

$$\frac{dB_{tot}}{dt}=\alpha_{3}B_{tot}\left( 1-B_{tot} \right)-\gamma_{1}LB_{tot}$$

$${-\gamma}_{2}AB_{tot}.$$

**Model J**

$$\frac{dL}{dt}=\alpha_{1}\frac{B_{tot}^{g_{3}}L^{g_{1}}}{(1+h_{2}A)}-\beta_{1}L,$$

$$\frac{dA}{dt}=\alpha_{2}\frac{B_{tot}A^{g_{2}}}{(1+h_{1}L)}-\beta_{2}A, (J)$$

$$\frac{dB_{tot}}{dt}=\alpha_{3}B_{tot}\left( 1-B_{tot} \right)-\gamma_{1}LB_{tot}$$

$${-\gamma}_{2}AB_{tot}.$$

**Models........**

**Model N-1**

$$\frac{dL}{dt}=\alpha_{1}B_{tot}^{g_{13}}{{(L}^{g_{1}}}/{(1+h_{2}A)})-\beta_{1}L,$$

$$\frac{dA}{dt}=\alpha_{2}B_{tot}^{g_{23}}{A^{g_{2}}}/{(1+h_{1}L)})-\beta_{2}A, (N-1)$$

$$\frac{dB_{tot}}{dt}=\alpha_{3}B_{tot}\left( 1-B_{tot} \right),-\gamma_{1}LB_{tot}-\gamma_{2}AB_{tot}$$

**Model N**

$$\frac{dL}{dt}=\alpha_{1}B_{tot}^{g_{13}}L^{g_{1}}A^{{-h}_{2}}-\beta_{1}A,$$

$$\frac{dA}{dt}=\alpha_{2}B_{tot}^{g_{23}}L^{{-h}_{1}}A^{g_{2}}-\beta_{2}A, (N)$$

$$\frac{dB_{tot}}{dt}=\alpha_{3}B_{tot}\left( 1-B_{tot} \right),-\gamma_{1}LB_{tot}-\gamma_{2}AB_{tot}$$

**Model Parameter Identifiability**.

We used the FME R package (*see* the methods section) to identify parameters that are correlated or that are collinear. A parameter set is said to be identifiable, if all parameters within the set can be uniquely estimated based on measurements [28]. Here we illustrate using Model A to show how this method can be applied to help detect parameters that are not highly correlated that can be fitted to data given a model. The rule of the thumb in this approach is to select a set of parameters with the collinearity index of less than 20 or with the least collinearity index. This method is only used to determine if the number of parameters predicted to explain a data set are not correlated. We did not use this method to determine the number of parameters to fit in the model because there could be few non-correlated parameters that will not fit the data.

# parameter sets $\alpha_{1}$ $\alpha_{2}$ $\alpha_{3}$ $g_{1}$ $\gamma_{1}$ # of parameters Collinearity index

1 1 1 0 0 0 2 1.0

2 1 0 1 0 0 2 1.1

3 1 0 0 1 0 2 16.1

4 1 0 0 0 1 2 1.2

5 0 1 1 0 0 2 1.0

6 0 1 0 1 0 2 1.0

7 0 1 0 0 1 2 1.0

8 0 0 1 1 0 2 1.1

9 0 0 1 0 1 2 4.5

10 0 0 0 1 1 2 1.3

11 1 1 1 0 0 3 1.1

12 1 1 0 1 0 3 16.1

13 1 1 0 0 1 3 1.2

14 1 0 1 1 0 3 18.3

15 1 0 1 0 1 3 6.0

16 1 0 0 1 1 3 17.8

17 0 1 1 1 0 3 1.1

18 0 1 1 0 1 3 4.5

19 0 1 0 1 1 3 1.3

20 0 0 1 1 1 3 5.9

21 1 1 1 1 0 4 18.3

22 1 1 1 0 1 4 6.0

23 1 1 0 1 1 4 17.8

24 1 0 1 1 1 4 18.5

25 0 1 1 1 1 4 5.9

26 1 1 1 1 1 5 18.5

A zero represents that a parameter will be excluded from a set of parameters with ones for the number of given parameters in a model to be fitted and obtain the given collinearity index. We note that 5 parameters from Model A have a collinearity index less than 20, therefore the 5 parameters are not highly correlated and can be used in model fitting.
